# An App-Based Mindfulness-Based Self-compassion Program to Support Caregivers of People With Dementia: Participatory Feasibility Study

**DOI:** 10.2196/28652

**Published:** 2021-11-26

**Authors:** Donna Goodridge, Nathan Reis, Jenna Neiser, Tim Haubrich, Bev Westberg, Laura Erickson-Lumb, Jo Storozinski, Cesar Gonzales, Joanne Michael, Allison Cammer, Nathaniel Osgood

**Affiliations:** 1 Department of Medicine University of Saskatchewan Saskatoon, SK Canada; 2 College of Kinesiology University of Saskatchewan Saskatoon, SK Canada; 3 Department of Computer Science College of Arts and Science University of Saskatchewan Saskatoon, SK Canada; 4 Saskatchewan Centre for Patient-Oriented Research Saskatoon, SK Canada; 5 Alzheimer Society of Saskatchewan Prince Albert, SK Canada; 6 Youville Centre Winnipeg, MB Canada; 7 Alzheimer Society of Saskatchewan Regina, SK Canada; 8 College of Pharmacy and Nutrition University of Saskatchewan Saskatoon, SK Canada; 9 Department of Computer Science Faculty of Arts and Science University of Saskatchewan Saskatoon, SK Canada

**Keywords:** virtual support programs, caregivers, dementia, mindfulness, self-compassion, mobile health, mobile applications, elderly, older adults, usability, feasibility, smartphone app, mobile phone

## Abstract

**Background:**

The number of persons with dementia is steadily growing, as is the number of individuals supporting persons with dementia. Primary caregivers of persons with dementia are most often family members or spouses of the persons with dementia, and they are more likely to experience increased stress and other negative effects than individuals who are not primary caregivers. Although in-person support groups have been shown to help buffer the negative impacts of caregiving, some caregivers live in isolated or rural communities and are unable to make the burdensome commitment of traveling to cities. Using an interdisciplinary approach, we developed a mobile smartphone support app designed for primary caregivers of persons with dementia, with the goal of reducing caregiver burden and easing stress. The app features a 12-week intervention, largely rooted in mindfulness-based self-compassion (MBSC), because MBSC has been linked to minimizing stress, depression, and anxiety.

**Objective:**

The primary objectives of our program are twofold: to explore the feasibility of a 12-week mobile support program and to conduct an initial efficacy evaluation of changes in perceived caregiver burden, coping styles, and emotional well-being of caregivers before and after the program.

**Methods:**

Our feasibility study used a 2-phase participatory pretest and posttest design, focusing on acceptability, demand, practicality, implementation, and efficacy. At phase I, we recruited 57 primary caregivers of persons with dementia (mean age 76.3, SD 12.9 years), comprising spouses (21/57, 37%), children (21/57, 37%), and friends or relatives (15/57, 26%) of persons with dementia, of whom 29 (51%) completed all measures at both pre- and postprogram. The content of the program featured a series of MBSC podcasts. Our primary outcome measure was caregiver burden, with secondary outcome measures including coping styles and emotional well-being. Daily ecological momentary assessments enabled us to ask participants, “How are you feeling today?” Phase II of our study involved semistructured follow-up interviews with most participants (n=21) who completed phase I.

**Results:**

Our findings suggest that our app or program meets the feasibility criteria examined. Notably, participants generally accepted the program and believed it could be a useful resource. Emotional well-being increased significantly (*P*=.04), and emotion-based coping significantly decreased (*P*=.01). Participants generally considered the app or program to be a helpful resource.

**Conclusions:**

Although there were no significant changes in caregiver burden, we were encouraged by the increased emotional well-being of our participants following the completion of our program. We also conclude that our app or program demonstrated feasibility (ie, acceptability, practicality, implementation, and efficacy) and can provide a much-needed resource for primary caregivers of persons with dementia. In the subsequent version of the program, we will respond to participant feedback by incorporating web-based weekly sessions and incorporating an outcome measure of self-compassion.

## Introduction

### Background

Dementia is a growing health concern that currently affects approximately 47 million people worldwide [1]. More than half of persons with dementia live in their own homes, supported primarily by family caregivers [2,3]. Family caregivers can face considerable stress when caring for a person with dementia at home [4,5], resulting in higher levels of depression and anxiety [6,7], social isolation [8], lower levels of subjective well-being [6-9] and worse physical outcomes for these individuals compared with caregivers of people without dementia [6-10]. One meta-analysis demonstrated caregivers of persons with dementia to be more stressed than nondementia caregivers and to experience more serious depressive symptoms and physical problems [6], whereas another found overall prevalence rates for depression and anxiety among primary caregivers of persons with dementia to be 34% and 44%, respectively, both figures being considerably higher than nondementia caregivers [11].

Although stress can often be alleviated through conventional education and counseling programs [12-14], participation in face-to-face interventions is not always feasible [15-17]. Caregiving responsibilities, the caregiver’s own health issues, the personal and economic burden of long travel distances to programs, and inclement weather can all pose major obstacles to program participation [16]. The COVID-19 pandemic has heightened awareness of the need to support caregivers of persons with dementia who were already at risk for social isolation before widespread precautionary restrictions were imposed [18].

To improve access to programs for caregivers who are not able to attend in person, the delivery of psychoeducational support programs through a mobile app is a promising, scalable solution. The ubiquitous nature of smartphones provides unprecedented opportunities for both content delivery and data collection. A systematic review [19] of mobile app-based health promotion programs, such as diet, physical activity, and lifestyle support, found better health outcomes for mobile app users compared with nonusers.

Although extensive research has focused on developing programs to alleviate burden in primary caregivers of persons with dementia [20,21], access to these programs remains limited and fragmented for many family caregivers [17-22]. Improving caregiver access to interventions may be enhanced through the judicious use of technology. A 2018 systematic review [15] of 8 randomized controlled trials of internet-based interventions for primary caregivers of persons with dementia concluded that the use of technology to teach people new coping skills to moderate stress can improve mental health, although marked methodological diversity prevented robust pooling of results.

For primary caregivers of persons with dementia, mindfulness-based interventions [23-27] have been shown to be more effective than traditional education and support. Self-compassion, a specific form of mindfulness training [28], is an approach to dealing with challenging or difficult situations that foster emotionally positive, understanding, and nonjudgmental attitudes toward oneself [29,30]. A systematic review [31] found that higher self-compassion in older adults was associated with lower levels of depression and anxiety and higher levels of well-being. The feasibility and effectiveness of delivering self-compassion programs on the internet has been demonstrated in several studies [32,33], but the proposed investigation is one of the first that we have identified to evaluate a mobile self-compassion program for primary caregivers of persons with dementia.

### Objectives

The overall objectives of this project are to: (1) explore the feasibility of a 12-week mobile support program, and (2) conduct an initial efficacy evaluation of changes in perceived caregiver burden, coping styles, and emotional well-being of caregivers before and after the program.

## Methods

### Design and Rationale

This feasibility study used a 2-phase participatory pretest and posttest design. Feasibility studies, which are considered particularly relevant to real-world settings, help to determine whether an intervention is appropriate for further testing and to identify the modifications in the research methods and protocols required [34]. Areas of focus for feasibility studies can include acceptability, demand, practicality, implementation, and efficacy. Acceptability refers to the extent to which a program is judged as suitable, satisfying, or attractive to participants, whereas demand can be demonstrated by interest or likelihood of use. Implementation is defined as the extent to which a new program can be successfully delivered to the intended audience [34]. Practicality refers to factors such as efficiency, speed, or quality of implementation and the ability of participants to undertake intervention activities [34]. A focus on limited efficacy involves the evaluation of whether the program shows promise of being successful, as examined by measuring the intended effects of the program on key intermediate variables [34].

Ethics approval for this study was obtained from the University of Saskatchewan (Behavioral Ethics Research Board #1014). Phase 1 involved the co-design of a mindfulness-based self-compassion (MBSC) intervention developed specifically for primary caregivers of persons with dementia. Our interdisciplinary team comprised researchers from the disciplines of nursing, nutrition, and computer science, as well as community representatives. Community advisors included one staff member from the Alzheimer Society of Saskatchewan (ASOS) and 2 patient family advisors (TH and BW). The advisory members helped to ensure all facets of the project reflected the values and interests of primary caregivers of persons with dementia and contributed to the ecological validity of the project [35]. Two certified MBSC consultants (JS and CG) with extensive experience working with primary caregivers of persons with dementia in the community participated in all team meetings, so that the MBSC content developed addressed the stated needs and preferences of the advisory members. This interdisciplinary approach drew upon complementary expertise and multiple perspectives to create an ecologically relevant program.

On the basis of caregivers’ lived experiences and preferences, the expertise of our self-compassion consultants and our review of the literature, the content for a 12-week MBSC and support program was co-designed within a 6-month time frame, incorporating existing web-based caregiver support resources developed by the ASOS or other reputable advocacy agencies (eg, Alzheimer’s Association), as appropriate. MBSC consultants developed the original material for this program in the form of 14 podcasts, 12 meditations, and 4 body-based practices. Table 1 describes the co-designed content categories and provides examples of the activities associated with each session.

**Table 1:** Co-designed app content.

Topics	Links to existing resources	Content example
Communication and dealing with difficult emotions	Difficult situations (eg, repetition and memory loss; wandering, paranoia) Difficult emotions (eg, guilt, anger, frustration)	Podcast “Who should Practice Mindfulness and Self-Compassion?”
Coping with stress, anxiety, and depression; Change and transition	Reducing caregiver stress Coping Overwhelm Rumination Emotional regulation YouTube video clip	Cognitive behavioral practice “Thought stopping”
Relationships, intimacy, and sexuality	Role changes, support, relationship dynamics, protection, loss, dealing with limited supports, boundaries	Meditation “Opening the Heart Space”
Grief and loss	Ambiguous loss and grief Anticipatory grief Loss of roles, relationships, independence, and history Changes in identity and personality	Meditations “Three Minute Breathing Space”
Caregiver fatigue and stress	Fatigue and exhaustion Stress Depletion Caregiver burden Respite Time for self-care and guilt	Podcast “Introduction to Loving Kindness Practice”
Positive ways to cope with caregiver fatigue and burn-out	Safety Insomnia and interrupted sleep Nutrition and activity	Body scan practice
More positive ways to cope with caregiver fatigue and burn-out	Developing resilience Gratitude Self-care	Podcast “The Power of Gratitude”

The program was delivered using the Ethica platform co-designed and codeveloped by the coauthor NO and Ethica Data Services. The Ethica platform is designed to aid in the creation, delivery, and data collection of smartphone-based apps [36,37]. A computer science student (JN) customized functionalities on the Ethica system to reflect the co-designed program.

Daily ecological momentary assessments (EMAs) sampled participants’ responses to the question “How are you feeling today?” The program offered a range of MBSC tools (including bespoke audio and video recordings and links to external resources such as relevant YouTube videos) with varying lengths between 1 and 20 minutes that caregivers could incorporate into their lives in ways that worked best for them [38]. Coping cards (eg, *Talk to yourself like you would to someone you love*) were developed to allow participants to access positive messages about coping and center them in a mindful and self-compassionate mindset.

Phase 2 consisted of a pre- and posttest design using validated instruments delivered through the app and qualitative data from postprogram individual interviews. The target sample size was 40 individuals. Information on demographic characteristics and current participation in support groups was collected after informed consent was obtained at enrollment. The Ethica app was installed on participants’ smartphones, and they received proper instruction on the use and privacy guarantees of the technology, including how to temporarily pause data collection. The duration of use data was gathered through the Ethica app use functionality. At the conclusion of 12 weeks or at termination of the program, participants were offered the opportunity to participate in individual telephone or Zoom interviews focusing on the experience of using the app.

### Participants

Caregivers were recruited by the ASOS using direct contact and social media, by linking the research team with ASOS caregiver programs in the province, and through media coverage and broadcast interviews. The eligibility criteria for this study included self-identification as a primary caregiver of a community-dwelling family member who has memory loss consistent with dementia, aged ≥18 years, able to read and speak English, and access to a smartphone. Participants received a CAD $100 (US $80.4) gift card to a grocery store of their choice to offset the data plan costs associated with using their personal devices during this study.

### Instruments

Caregiver burden, the primary outcome, was measured using the Burden Scale for Family Caregiving (BSFC) [39] at baseline and 1 week after the conclusion of the program through the Ethica app. The BSFC is a widely used 28-item questionnaire developed to identify individual caregiver service needs, predict caregiver health in research studies, and evaluate the effectiveness of programs [40,41]. The degree of subjective burden was expressed as the level of disagreement with the statements. Furthermore, 12 of the 28 items were inversely presented to minimize the potential for response bias. The completion of the BSFC takes approximately 5-10 minutes. The possible scores ranged from 0 to 84. The BSFC cumulative score is assigned to three levels of subjective burden categories specific to caregivers of persons with dementia: none to mild (0-35), moderate (36-45), and severe (46-84) [42]. Standardization with 1911 subjects established the average BSFC score for caregivers of persons with dementia as 41.9 (SD 14.8); Cronbach α was .92, and test-retest reliability was 0.94 [39].

The secondary outcomes included changes in coping style and emotional well-being. Short questionnaires were delivered daily via the app to the participants on a rotating basis. One 2-item scale from the Brief-COPE instrument [43,44] was delivered every day. The Brief-COPE is a validated, 28-item measure of different coping styles comprising 14 2-item scales [43,44]. Coping styles refer to an individual’s response to a psychological stressor, which is often related to a negative event [43]. These styles include self-distraction, active coping, denial, substance use, use of emotional support, use of instrumental support, behavioral disengagement, venting, positive reframing, planning, humor, acceptance, religion, and self-blame. Total scores were calculated for each scale, allowing us to detect whether changes in coping style occurred over the duration of the program [44]. These scales can be grouped into emotion-focused, problem-focused, and dysfunctional coping styles. Emotion-focused coping styles aim to reduce, alleviate, and/or minimize the unpleasant feelings associated with the stressor and are especially valuable in situations in which the person has little control [44], as can often occur in caring for a person with dementia.

In addition to the Brief-COPE, the World Health Organization (WHO)-5 Well-Being Scale was completed weekly. The WHO-5 is a short, commonly used, psychometrically sound measure of positive emotional well-being with a single cumulative score, where 100 represents the best possible quality of life [45].

### Qualitative Data

This paper summarizes data from field notes kept by research assistants during enrollment and program delivery, as well as interview data related specifically to the technical aspects of the app in terms of acceptability, practicality, and implementation for the 72% (21/29) of participants included in the quantitative analyses who also completed the final interviews.

### Data Analysis

Pearson correlations were used to assess the relationships between raw scores on the BFSC and personal characteristics (continuous variables), WHO-5 scores, and the three Brief-COPE (emotion-focused, problem-focused, and dysfunctional coping styles) summary scores. Descriptive analyses were performed to detect differences in scores at baseline and at the end of the program on the key variables of interest (ie, burden, coping styles, and well-being). Wilcoxon signed-rank tests were used to compare baseline and final scores on the BSFC, the WHO-5, and the three (ie, emotion-focused, problem-focused, and dysfunctional coping styles) summary scores on the Brief-COPE. A *P* value of <.05 was considered as statistically significant. All statistical analyses were conducted using the SPSS version 27 (IBM Inc). Content analysis [46] using a deductive approach allowed for the targeted analysis of qualitative data related to acceptability, practicality, and implementation.

## Results

### Participants

A total of 77 participants were enrolled in the study, although 16 did not open the app. One of these individuals indicated: *“*I’m past tired now without reading stuff first to download [the app]...even simple instructions are too much.*”* Three participants withdrew after several weeks because of busy personal schedules or other priorities and another was found not to own a smartphone.

A total of 53 participants (48/53, 91% female) with a mean age of 58.0 (SD 13.6) years were recruited into the study between September 2019 and March 2020. The persons with dementia for whom participants were caring included spouses (19/53, 36%), parents (17/53, 32%), and other friends or relatives (17/53, 32%) with a mean age of 77.6 (SD 12.0) years. Complete baseline and final data sets (BFSC, WHO-5, and Brief-COPE) were available for 51% (29/57) of participants who completed all questionnaires at both baseline and the end of the program and were included in the data analysis below. Demographic characteristics of excluded participants were compared using 2-tailed *t* tests (age) and chi-square analyses (participant sex, care recipient sex, and age), and no significant differences were detected.

Table 2 displays the demographic and personal characteristics of the participants. The participants were mostly female (26/29, 90%), with excellent or very good health (18/29, 62%) with a mean age of 59.6 (SD 11.3; range 28-79) years. Most participants cared for a spouse or parent (22/29, 76%). The mean age of care recipients was 78.9 (SD 10.1) years.

**Table 2:** Demographic and personal characteristics (N=29).

Characteristics		Value, n (%)
**Gender**		
	Female	26 (90)
	Male	3 (10)
**Relation to persons with dementia**		
	Spouse	11 (38)
	Child	11 (38)
	Other relative	6 (21)
	Friend	1 (3)
**Currently work for pay**		
	Yes	11 (38)
	No	18 (62)
**Have one or more family members available for support**		
	Yes	24 (83)
	No	5 (17)
**Duration of caregiving for persons with dementia**		
	2 years or less	13 (45)
	3-6 years	7 (24)
	7 years or more	9 (31)
**Participant self-rated health**		
	Excellent	4 (14)
	Very good	14 (48)
	Good	9 (31)
	Fair	2 (17)
	Poor	0 (0)
**Rating of persons with dementia health**		
	Excellent	0 (0)
	Very good	4 (14)
	Good	5 (17)
	Fair	16 (55)
	Poor	4 (14)
**Persons with dementia behaviors reported**		
	Memory loss	28 (97)
	Refusing help	16 (55)
	Repetitive behaviors	15 (52)
	Sleep disturbances	13 (45)
	Paranoia	10 (34)
	Hoarding	8 (28)
	Aggression	6 (21)
	Wandering	6 (21)
	Other	10 (34)
**Total number of behaviors reported**		
	1-3	13 (45)
	4 or more	16 (55)

### Acceptability, Practicality, and Implementation of the App

Understanding the acceptability, practicality, and implementation of using an app to deliver a psychoeducational program targeted at caregivers of persons with dementia was central to this study, especially because of the wide variation in possible caregiver ages and comfort with technology. Participants generally found the app easy to use and user-friendly, although several required additional assistance from the research assistant to address navigation problems early in the program. One participant noted:

I’m not a very techy person, so I was really nervous about it at first...But anyway, once I got that it was fine.

Many participants noted the convenience of having the content available on their phones:

I liked that it was on my phone. I liked that I could access it at my convenience. And I do use my phone pretty much like my right hand all the time.

The availability of multiple types of content was very appealing to many participants:

It’s got a variety of different things in one place. I like that. It’s like a one stop shop.

The incorporation of EMAs has received numerous favorable comments:

The most useful part was the, “How are you feeling,” survey, every day. It made me sit down and think about the last few hours, and was how stressed I really was, or maybe I wasn’t as stressed as I thought I was. And I’ve come to look forward to that, so I actually have a minute to sit down and say, “Well how was just the last bit?” I found that really, really helpful.

Incorporation of appropriate YouTube videos was appreciated by most participants, but several participants did not like the automatic redirection to alternate videos that were not part of the program. Several commented that they wished that the program could also be available on their computers to improve readability.

Over the 12 weeks of the program, participants’ mean hours of app use were 15.60 (SD 28.83) hours with a median time of 5.31 hours (IQR 3.0-11.1).

### Outcome Measures

As there was minimal variability in the BFSC, WHO-5, and Brief-COPE subscale scores obtained midprogram and at the end of the program, this study compared only baseline and the final scores obtained on these instruments.

At baseline, the median BFSC score was 45 (IQR 35.5-50; range 31-61). Most participants were categorized as having either no to mild burden (12/29, 41%) or moderate burden (16/29, 55%), with 3% (1/29) reporting severe burden. Table 2 displays the demographic characteristics of the participants according to burden categories. BFSC scores were negatively correlated with emotional well-being (*r*=−0.40; *P*=.03) and positively correlated with the use of avoidance-based coping (*r*=0.57; *P=*.001) and the number of behaviors exhibited by the persons with dementia (*r*=0.42; *P*=.02). There were no correlations between BFSC scores and caregiver age (*r*=−0.17; *P*=.39), duration of providing care (*r*=0.30; *P*=.11); age of persons with dementia (*r*=0.10; *P*=.60), emotion-focused coping (*r*=0.08; *P*=.70), and problem-focused coping (*r*=0.07; *P*=.73).

Table 3 displays the frequencies of the coping strategies reported by the participants at baseline. Emotion-focused coping strategies, particularly acceptance, were most frequently used by participants at baseline. The two acceptance items, “I’ve been accepting the reality of the fact that it has happened” and “I’ve been learning to live with it,” were used by 52% (15/29) and 45% (13/29), respectively. Almost half (14/29, 48%) also reported frequent use of the problem-focused coping strategy, “I’ve been thinking hard about what steps to take.” Two avoidance and dysfunctional coping strategies were reported by about one-third of participants: “I’ve been blaming myself for things that happened” (11/29, 38%) and “I’ve been turning to work or other activities to take my mind off things” (9/29, 31%). There was minimal endorsement of other avoidance and dysfunctional coping strategies.

**Table 3:** Frequencies of reported baseline coping strategies (N=29).

Coping strategy			Frequency of use, n (%)						
			Not at all		A little bit		Medium amount		A lot
**Emotion-based coping strategies**									
	I have been trying to see it in a different light, to make it seem more positive.	2 (7)		11 (38)		11 (38)		5 (17)	
	I have been getting comfort and understanding from someone.	3 (10)		10 (34)		5 (17)		11 (38)	
	I have been looking for something good in what is happening.	7 (24)		8 (28)		9 (31)		5 (17)	
	I have been making jokes about it.	16 (55)		7 (24)		3 (10)		3 (10)	
	I have been accepting the reality of the fact that it has happened.	4 (14)		2 (7)		9 (31)		15 (52)	
	I have been trying to find comfort in my religion or spiritual beliefs.	11 (38)		4 (14)		3 (10)		11 (38)	
	I have been learning to live with it.	0 (0)		3 (10)		13 (45)		13 (45)	
	I have been praying or meditating.	9 (31)		4 (14)		5 (17)		11 (38)	
	I have been making fun of the situation.	21 (72)		7 (24)		0 (0)		1 (3)	
**Problem-based coping strategies**									
	I have been concentrating my efforts on doing something about the situation I am in.	2 (7)		9 (31)		9 (31)		9 (31)	
	I have been getting emotional support from others.	5 (17)		13 (45)		8 (28)		3 (10)	
	I have been taking action to try to make the situation better.	0 (0)		10 (34)		11 (38)		8 (28)	
	I have been getting help and advice from other people.	3 (10)		15 (52)		10 (34)		1 (3)	
	I have been trying to come up with a strategy about what to do.	3 (10)		10 (34)		5 (17)		11 (38)	
	I have been trying to get advice or help from other people about what to do.	3 (10)		18 (62)		6 (21)		2 (7)	
	I have been thinking hard about what steps to take.	1 (3)		6 (21)		8 (28)		14 (48)	
**Avoidance and dysfunctional coping strategies**									
	I have been turning to work or other activities to take my mind off things.	6 (21)		7 (24)		7 (24)		9 (31)	
	I have been saying to myself “this isn’t real.”	17 (59)		8 (28)		2 (7)		2 (7)	
	I have been using alcohol or other drugs to make myself feel better.	21 (72)		3 (10)		4 (14)		1 (3)	
	I have been giving up trying to deal with it.	16 (55)		8 (28)		2 (7)		3 (10)	
	I have been refusing to believe that it has happened.	21 (72)		4 (14)		4 (14)		0 (0)	
	I have been saying things to let my unpleasant feelings escape.	9 (31)		11 (38)		6 (21)		3 (10)	
	I have been using alcohol or other drugs to help me get through it.	22 (76)		5 (17)		2 (7)		0 (0)	
	I have been criticizing myself.	2 (7)		11 (38)		10 (34)		6 (21)	
	I have been giving up the attempt to cope.	17 (59)		11 (38)		0 (0)		1 (3)	
	I have been doing something to think about it less, such as going to movies, watching television, reading, daydreaming, sleeping, or shopping.	2 (7)		11 (38)		11 (38)		5 (17)	
	I have been expressing my negative feelings.	3 (10)		15 (52)		9 (31)		2 (7)	
	I have been blaming myself for things that happened.	9 (31)		4 (14)		5 (17)		11 (38)	

Table 4 displays the demographic characteristics of the participants according to burden categories. BFSC scores were negatively correlated with emotional well-being (*r*=−0.40; *P*=.03) and positively correlated with the use of avoidance-based coping (*r*=0.57; *P*=.001) and the number of behaviors exhibited by the persons with dementia (*r*=0.42; *P=*.02). There were no correlations between BFSC scores and caregiver age (*r*=−0.17; *P*=.39), duration of providing care (*r*=0.30; *P*=.11); age of persons with dementia (*r*=0.10; *P*=.60), emotion-focused coping (*r*=0.08; *P*=.70), and problem-focused coping (*r*=0.07; *P*=.73).

**Table 4:** Burden ratings and participant and care recipient characteristics (N=29).

Characteristics		None to mild burden (0-41; n=12), n (%)	Moderate to severe burden (≥42; n=17), n (%)
**Relationship to persons with dementia**			
	Spouse	7 (64)	4 (36)
	Parent	2 (18)	9 (82)
	Other	3 (43)	4 (57)
**Currently work for pay**			
	Yes	3 (27)	8 (73)
	No	9 (50)	9 (50)
**Caregiver self-rated health**			
	Excellent or very good	9 (50)	9 (50)
	Good, fair, or poor	3 (27)	8 (73)
**Persons with dementia health**			
	Excellent, very good, or good	6 (67)	3 (33)
	Fair or poor	6 (30)	14 (70)

Table 5 compares the baseline and end-of-program scores for the outcome measures. No significant change was detectable in the moderate level of caregiver burden reported by the participants following this intervention. A statistically significant (*P=*.04) increase in emotional well-being as measured by the WHO-5 was noted. No differences were evident in the Brief-COPE scores for problem-based or avoidance and dysfunctional coping, but the decrease in emotion-focused coping was statistically significant (*P=*.01).

**Table 5:** Comparison of baseline and end-of-program scores.

Outcome	Baseline median (IQR)	End median (IQR)	WS-R^a^ (*Z* score)	*P* value
Burden scale	45 (35.5-50)	42 (35-48)	−0.56	.57
WHO-5^b^	52 (28-72)	52 (32-80)	−2.09	.04^c^
Brief-COPE emotion-based coping	18 (15-22)	15 (13-18.5)	−2.49	.01^c^
Brief-COPE problem-based coping	18 (15-22)	18 (15-21.5)	−0.45	.66
Brief-COPE avoidance and dysfunctional coping	21 (18-15.5)	21 (17.5-26.5)	−0.55	.59

^a^Wilcoxon signed rank.

^b^WHO-5: World Health Organization-5.

^c^Difference statistically significant at *P*<.05.

## Discussion

### Principal Findings

This study examined 4 aspects of feasibility (acceptability, practicality, implementation, and efficacy) in relation to a co-designed web-based support program for primary caregivers of persons with dementia delivered via an app on a smartphone. Our deployment results revealed that participants valued the *one-stop shop* approach of having a range of MBSC practices (acceptability) available on their personal smartphones (practicality) that could be used to support them in their caregiving challenges. A few technical problems were experienced, and the app was considered easy to use (implementation). This is especially important in studies such as this, where participants are anticipated to lack the high comfort and smartphone skills (ie, digital literacy) of digital-native generations [47].

The interdisciplinary collaboration between health care researchers and computer scientists afforded a unique opportunity to capitalize on the expertise of multiple disciplines and to deploy a program in a reasonably short window. Although each discipline has its own unique body of knowledge and jargon, ongoing discussions between team members allowed all the voices to be heard in a respectful manner, to achieve consensus on key aspects of the project, and to undertake a study that no one team member could have achieved without such a collaboration.

The participatory, co-design approach to this project ensured that the perspectives of key stakeholders such as primary caregivers of persons with dementia and the Alzheimer’s Society were incorporated into content and program development, which we consider a strength of our project. The value of co-designed programs has been amply demonstrated in the literature, notably in the field of technological support for chronic diseases and/or ailments, given the purposes of our research [48,49]. Despite some challenges that can stem from co-designed programs (eg, time issues can interfere with a fully democratic process [49]), they remain the gold standard for program development in the field of integrated care [50]. The advantages of co-designed support apps such as ours include knowledge cocreation across technical solutions, lived experience, and medical expertise [48] and the reduction of social health inequalities [50]. For caregivers of functionally dependent adults like many of the primary caregivers of persons with dementia in our research, co-designed support apps have shown value by enabling caregivers to identify their needs and tailoring the support program accordingly [49]. Moreover, inclusivity and feasibility are enhanced through co-designed programs because users and/or stakeholders can provide insight into important considerations, including appropriate digital literacy levels and respect for the help-seeking process of users [50].

By incorporating perspectives of primary caregivers of persons with dementia and members of the Alzheimer’s Society in the development process of our support program, we adhered to the guiding principle of integrated care [50]. In addition, by collecting feedback from the primary caregivers of persons with dementia in the form of semistructured interviews post program completion, the co-design approach will be built into future iterations of the program or app design. Overall, participants and invested stakeholders (eg, members of the Alzheimer’s Society) shaped this version of our program and offered suggestions to improve it moving forward. It is our view that committing to a user-centered support program promotes optimization of the final product, at least in part, through the enhanced acceptance, usability, and feasibility of its users.

The content and data collection instruments were successfully delivered via the smartphone as planned, but only 51% (29/57) of participants completed all questionnaires. Although data collection using smartphones offers the advantages of ecological validity and real-time data, missing data in these types of studies is a well-recognized problem [47]. The participant burden of completing the data collection protocol, which involved multiple administrations of 3 questionnaires throughout the 12-week program, likely contributed to missing data in this sample of caregivers and was noted by some of the participants. Future studies with primary caregivers of persons with dementia delivering programs via smartphones should consider whether interviewer-administered data collection can be integrated into studies.

Evaluation of the short-term efficacy of this intervention to support primary caregivers of persons with dementia yielded mixed results. During this feasibility study, participants chose their own level of engagement with the content of the program, and the hours spent on the content varied widely, as did the content that was accessed by participants. As this study sought to establish the feasibility of delivering this program via smartphones, the duration of the program and follow-up period were relatively constrained.

No change in caregiver burden scores was detectable immediately following the program, which may be attributable to several factors. As caregiver burden is influenced by diverse factors [51], including the cognitive function of persons with dementia, hours spent caregiving, the caregiver’s level of social support, and previous caregiver experience, any positive impact of beginning to incorporate an MBSC approach may have been overshadowed by these other factors. We encourage future researchers to examine whether some of these exogenous factors may impede the efficacy of MBSC programming. In addition, the relatively short duration of the program may have contributed to the lack of change in burden.

As there was no significant reduction in caregiver burden, our primary outcome measure, from baseline to post intervention (*P*=.57), we conducted a post hoc reliable change index analysis in an effort to detect individual changes. The results of the reliable change index showed that 7 participants had a statistically significant reduced burden, whereas 1 participant had a significantly increased burden from baseline to post intervention.

No differences were detected in problem-based or avoidance-dysfunctional coping styles, but there was an unexpected decrease in emotion-based coping. We suspect that a variety of factors contributed to this decrease in emotion-based coping following our intervention. For instance, diminished health of persons whom our participants were caring for might have made it more difficult for caregivers to engage in emotion-focused coping behaviors that make light of the situation, like “I’ve been making jokes about it.” Some of our participants noted in the follow-up interviews that the health of their loved ones deteriorated significantly over the course of the 8-week program, which could have been partly responsible for the unexpected decrease in emotion-based coping approaches. The introduction to MBSC approaches in this study could potentially strengthen cognitive restructuring as a coping strategy but has not been identified as affecting the degree to which people use problem solving or avoidance and dysfunctional coping [52].

Emotional well-being of participants showed a small, but statistically significant improvement, although this failed to meet the 10% change in scores recommended to signify a *clinically* significant difference. Around 48.3% (14/29) of participants scored lower than 50 on the WHO-5 and met the criteria for screening for depression, highlighting that caregivers are at risk for adverse emotional sequelae.

### Strengths and Limitations

Our study enabled primary caregivers of persons with dementia to access resources from the convenience of their smartphones that they may not otherwise have been able to use, especially considering that some of the content was developed specifically for this project (eg, MBSC podcasts). Although it is possible that some of the primary caregivers of persons with dementia could attend in-person support groups where similar content could be available, others cited their geographic isolation in remote communities or rural settings as a barrier to doing so, and that having the material so readily available to them was particularly helpful, as was the user-friendly nature of our app and content. In addition, with current COVID-19 restrictions, in-person support group meetings may be less prevalent in various locations.

One potential shortcoming of our research is rooted in the nature of feasibility studies, as not all possible outcomes can be measured. To reduce the burden on respondents, we did not measure changes in MBSC, which have typically been evaluated using the Self-Compassion Scale (SCS) [53]. However, given that there might be a connection between MBSC and reduced caregiver burden, we recommend future programs including the SCS or perhaps its short-form.

Because of missing data, only half of the questionnaires could be included in the final analyses, although we found no differences in the demographic characteristics of those who completed all questionnaires and those who did not.

The experimental nature of our study ensured that we assessed measures pre- and postintervention. However, a control group was not included in our study, which can be considered a limitation. The rationale behind our decision to not include a control group was to ensure that participants were not deceived, particularly given their vulnerable status as primary caregivers of persons with dementia, and also to better assess the feasibility of our program by enrolling all participants in it. Ultimately, we wanted to know if the core elements of our program were well received by participants, although we acknowledge that it might be advisable for future iterations to include a control group. In addition, a 1-month postintervention follow-up assessment is recommended to researchers to examine whether intervention effects can withstand the test of time. In this study, we interviewed participants approximately 1 month after they completed the program to collect their thoughts on its acceptability, implementation, and feasibility, although quantitatively assessing primary outcome measures would be advantageous.

An additional limitation is that the vast majority of participants were female (26/29, 90%), meaning that although the program was received favorably overall, its generalizability is somewhat unknown. For instance, the male primary caregiver of persons with dementia might reject our program initially or they might not find it effective. However, there was male representation among our patient family advisors (ie, TH), though perhaps future iterations of the program should be informed by a more equal representation of males and females across varying age groups.

### Future Directions

Our program as currently structured has value and utility for primary caregivers of persons with dementia, as evidenced by our results and in postintervention interviews with our participants. However, we acknowledge ways that our program could be improved in future iterations, starting with usability. Specifically, some participants expressed that they felt burdened by certain inclusions (eg, daily EMAs), whereas others valued these inclusions. Accordingly, we recommend that future versions have optional EMAs, for example, where participants can choose the number of daily EMAs (eg, maximum of 2/day, minimum of 2/week, for instance). We also encourage researchers to use a version of our program to include a measure of MBSC—specifically, the SCS—at each time point, as a way to determine whether mindful self-compassion levels increase following the MBSC intervention.

Other considerations for future iterations of our program include weekly web-based meetings among small groups of participants via breakout rooms, to enhance a sense of community and/or support that was lacking in the current version. These breakout rooms could be moderated by an *expert* in the field from the research team to ensure that participants’ questions were addressed or answered. In addition, a postintervention focus group *wrap up* session could be delivered virtually, where the primary caregivers of persons with dementia could connect with the experts and one another. Finally, as communicated by participants, more coping cards and a clearer layout of each week’s content should be introduced.

As noted in a thematic literature analysis by Rampioni et al [54], collaboration between researchers, technology developers, patients, and caregivers remains a significant challenge in developing technologies appropriate to support dementia. Our future directions include sustaining the interdisciplinary, community-involved team we have developed and potentially expanding the team to include additional disciplines such as psychiatry and social work.

### Conclusions

Our study is one of the first to co-design and deliver an MBSC program for caregivers of persons with dementia using a smartphone. We believe that the findings of this study have demonstrated the feasibility and demand for this type of web-based program and identified the key challenges to be addressed in future studies. Specifically, it is our view that an MBSC program for primary caregivers of persons with dementia, like the one presented in this study, can be particularly helpful for individuals in rural or isolated communities, with limited access to support groups. In addition, this feasibility study has helped identify key outcome variables that were left out of this version (eg, self-compassion), while also enabling us to address intervention elements that can be altered (eg, daily EMAs) or incorporated in the next installment of our intervention (eg, web-based breakout groups to develop both a sense of community among primary caregivers of persons with dementia lacking in the current iteration and to enable participants to ask questions as they progress through the intervention). Our findings will inform the development of future iterations of the MBSC program and will contribute to the evidence on strategies to better support caregivers of persons with dementia.

## Abbreviations

ASOS: Alzheimer Society of Saskatchewan
BSFC: Burden Scale for Family Caregiving
EMA: ecological momentary assessment
MBSC: mindfulness-based self-compassion
SCS: Self-Compassion Scale
WHO: World Health Organization
